# Left colic artery aneurysm rupture after stent placement for abdominal aortic aneurysm associated with neurofibromatosis type 1

**DOI:** 10.1186/s40792-019-0570-4

**Published:** 2019-01-23

**Authors:** Kazuki Moro, Hitoshi Kameyama, Kaoru Abe, Junko Tsuchida, Yosuke Tajima, Hiroshi Ichikawa, Masato Nakano, Mayuko Ikarashi, Masayuki Nagahashi, Yoshifumi Shimada, Kaori Kato, Takeshi Okamoto, Hajime Umezu, Emmanuel Gabriel, Masanori Tsuchida, Toshifumi Wakai

**Affiliations:** 1 Division of Digestive and General Surgery, Niigata University Graduate School of Medical and Dental Sciences, 1-757 Asahimachi-dori, Chuo-ku, Niigata City, Niigata 951-8510 Japan; 2 Division of Thoracic and Cardiovascular Surgery, Niigata University Graduate School of Medical and Dental Sciences, 1-757 Asahimachi-dori, Chuo-ku, Niigata City, Niigata 951-8510 Japan; 3 Division of Molecular and Diagnostic Pathology, Niigata University Graduate School of Medical and Dental Sciences, 1-757 Asahimachi-dori, Chuo-ku, Niigata City, 951-8510 Japan; 4 Department of Surgery, Mayo Clinic, Jacksonville, FL USA

**Keywords:** Aneurysm, Left colic artery, Left hemicolectomy, Neurofibromatosis type 1, von Recklinghausen disease

## Abstract

**Background:**

Neurofibromatosis type 1 (NF1) is an autosomal dominant disease of the skin and soft tissue. Aneurysms associated with NF1 can occur, but a secondary aneurysm rupture is very rare, with very few cases reported in literature.

**Case presentation:**

We describe the case of a 67-year-old female with NF1 who underwent endovascular aneurysm repair (EVAR) for an abdominal aortic aneurysm (AAA) rupture. She developed a type Ib endoleak requiring a redo-EVAR. Eighteen days after her primary operation, she was found to have two new left colic artery aneurysms. She required emergency surgery consisting of a left hemicolectomy and transverse colon colostomy. Pathology showed neurofibromatous changes to the peri-vasculature tissue, consistent with her underlying disease.

**Conclusions:**

Although rare, secondary aneurysms can occur following AAA repair. Patients with soft tissue connective tissue disorders, like NF1, may be at an increased risk for development of these secondary aneurysms. Endovascular repair appears to be a safe approach for NF1 patients with AAA, but endovascular management can be challenging in the setting of NF1. Surgeons should be ready to convert to open surgery if the patient displays persistent signs of bleeding or structural changes related to connective tissue disorders like NF1.

## Background

Neurofibromatosis type 1 (NF1), also known as von Recklinghausen disease, is an autosomal dominant disease of the skin and soft tissue that occurs in approximately 1 in 2500–3000 people [1, 2]. NF1 is caused by a genetic mutation in the NF1 gene, located on the long arm of chromosome 17 (17q11.2) [3]. Typical signs of NF1 include café-au-lait macules [4], skinfold freckling [5], neurofibromas [6], brain tumors [7], and characteristic bony lesions [8]. Moreover, patients with NF1 are at increased risk for scoliosis [9], vascular abnormalities [10], and cancer [11], including breast cancer [12].

Vascular abnormalities associated with NF1 occur in 0.4–6.4% of patients [13, 14] and most commonly involve the aorta, renal arteries, mesenteric arteries, and visceral arteries [15, 16]. There are some reports describing aortic aneurysms with NF1 [17], but secondary aneurysm rupture of the left colic artery (LCA) is a rare event, with few cases reported in the literature. In this case report, we describe our surgical experience with a brief review of other reported cases in the literature.

## Case presentation

**Fig. 1:**
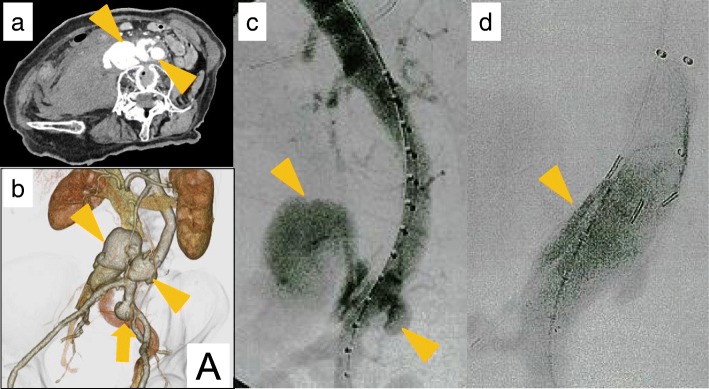
Images of the abdominal aortic aneurysm before and during the primary operation. **a** A computed tomography (CT) scan of the abdomen and pelvis demonstrated rupture of the abdominal aortic aneurysm (orange arrow head). **b** A 3D-CT scan reconstruction of the abdomen and pelvis demonstrated the abdominal aortic aneurysm with a fistulous communication between the aneurysm and the inferior vena cava (orange arrowhead) and the left common iliac artery aneurysm (orange arrow). **c** Arterial angiography demonstrated the abdominal aortic aneurysm with fistulous communication between the aneurysm and the inferior vena cava (orange arrowhead). The location of aorta rupture was located 7.5 cm distal to the renal arteries and 2.5 cm proximal to the bifurcation of the aorta. **d** We deployed an ENDURANTII iliac extension far proximal to the terminal aorta that was long enough to place three or more stents (orange arrowhead)

A 67-year-old female with NF1 presented with lumbago, cold sweats, and sudden onset weakness, which necessitated an emergency transfer to our institution. She had no significant past medical history, was negative for hypertension, and was on no regular medications. She was hypotensive (systolic blood pressure = 60 mmHg) and tachycardic on arrival. An abdominal contrast computed tomography (CT) scan showed a rupture of an abdominal aortic aneurysm (AAA) with communication between the aneurysm and the inferior vena cava (IVC) (Fig. 1a, b). The maximum dimensions of the ruptured AAA and IVC were 34 mm and 37 mm, respectively. The aortic rupture was located 7.5 cm distal to the renal artery and 2.5 cm proximal to the bifurcation of the aorta (Fig. 1c). Due to the difficulty of primarily closing the ruptured IVC, we planned an endovascular treatment to control the bleeding from the IVC by exclusion of the ruptured AAA. The diameter of normal proximal aorta was 16 mm, which was too narrow to place a normal Y-shaped graft. Moreover, there was insufficient time to prepare another stent in emergency. Therefore, we instead deployed an ENDURANTII (Medtronic Vascular, Santa Rosa, CA, USA) iliac extension proximal to the terminal aorta that was long enough to insert three or more stents (Fig. 1d). As the bleeding from the AAA and the communication between the aneurysm and the IVC were not well controlled, we placed an EXCLUDER (W.L. Gore & Associates, Flagstaff, AZ, USA) cuff in the ENDURANTII iliac extension. Although a type IV endoleak was detected on angiography, the patient’s hemodynamics stabilized. We therefore decided to conclude the operation at this point and re-assess the endoleak in a few days.

**Fig. 2:**
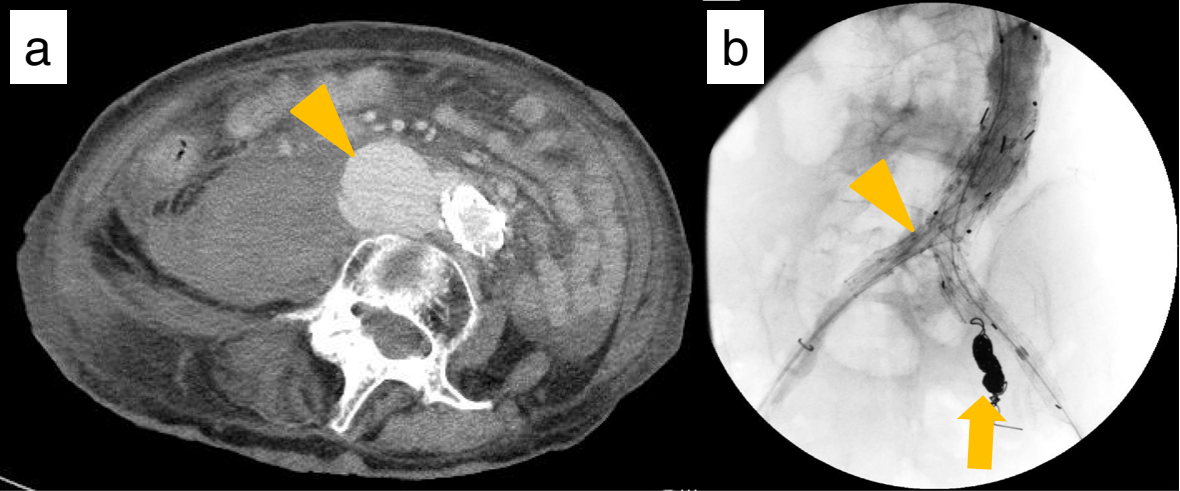
Images of the abdominal aortic aneurysm before and during the secondary operation. **a** An abdominal contrast CT demonstrated type Ib endoleak (orange arrowhead). **b** An AFX stent and infrarenal cuff (orange arrowhead) were deployed, and a coil embolization performed of the left internal iliac artery aneurysm (orange arrow)

An abdominal contrast CT performed 3 days after the operation showed a type Ib endoleak and injury to the distal abdominal aorta (Fig. 2a). This led to a redo-EVAR 4 days after operation. This included deploying an AFX (Endologix, Inc., Irvine, CA, USA) graft, as well as an infrarenal cuff, as the AFX head did not fit exactly within the stent placed in the primary operation. We also performed coil embolization treatment of the left internal iliac artery aneurysm (Fig. 2b), which was identified initially (Fig. 1b). We confirmed no endoleak at the final angiography. An abdominal contrast CT performed 18 days after the primary operation showed two new sequential aneurysms of the LCA, which were not previously detected. Coil embolization was planned to address these new aneurysms.

**Fig. 3:**
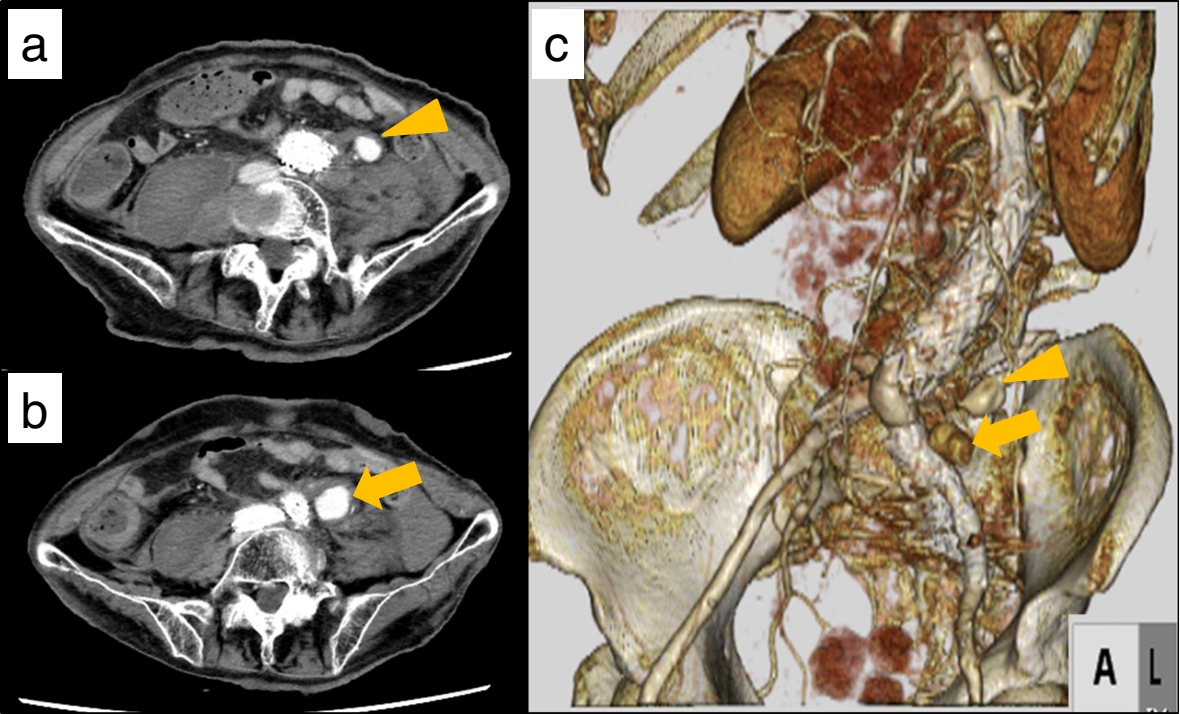
Radiographic images of the left colic artery aneurysm prior to the patient’s re-operation. **a**, **b** A CT scan of the abdomen and pelvis demonstrated the distal (**a**) (orange arrowhead) and proximal (**b**) portions of the left colic artery aneurysm (orange arrow). **c** A 3D-CT scan reconstruction of the abdomen and pelvis demonstrated the two locations of the left colic artery aneurysms (orange arrowhead and orange arrow)

Twenty-two days after primary operation, however, the patient presented with new onset nausea, left abdominal pain, and hypotension. Her systolic blood pressure was 50 mmHg. She was resuscitated and required vasopressor support. Laboratory tests, including leukocyte count and electrolytes, were normal. The serum C-reactive protein and D-dimer were elevated to 0.37 mg/dl (normal range 0.01–0.30) and 6.8 μg/ml (normal range 0.0–1.0), respectively. We considered potential bleeding from nearby arteries, including the inferior mesenteric artery (IMA) and lumbar artery [18, 19]. An abdominal contrast CT showed enlargement of LCA aneurysms and surrounding hematoma (Fig. 3a–c), establishing the diagnosis of rupture of the LCA aneurysms. Emergency exploratory surgery was performed.

**Fig. 4:**
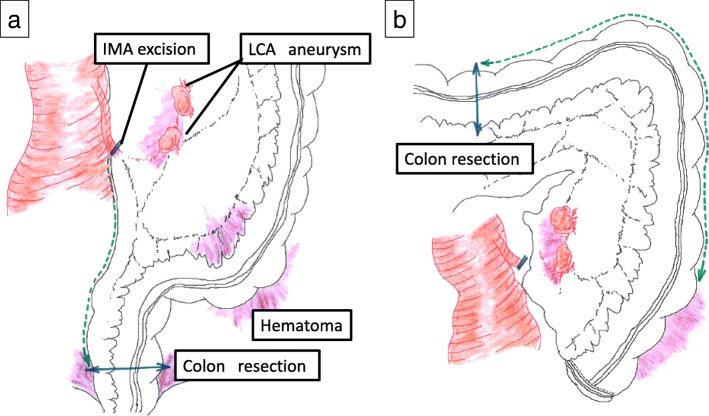
Surgical approach to the left colon and mesentery containing the left colic artery (LCA) aneurysms. **a** We divided the inferior mesenteric artery (IMA) 3 cm distal to the IMA root so as to not to injure the aorta. We divided the colon at the rectosigmoid junction. **b** We divided the middle transverse colon and included the distal transverse colon as part of the colectomy

We gained access through a midline abdominal incision. Active bleeding was encountered intraperitoneally and controlled with compression. As a result of the patient’s initial AAA rupture, residual hematoma was present, which extended along the entire left abdomen and retroperitoneum (Fig. 4a). In addition, the AAA stent endograft had resulted in a left laterally displaced aorta, narrowing our operative field. Due to her distorted anatomy, active bleeding, and clinical condition, we elected to perform a left hemicolectomy. We divided the inferior mesenteric artery (IMA) 3 cm distal to the IMA root to not to injure the recently repaired aorta. We divided the colon at the rectosigmoid junction. Proximally, we divided the colon at the middle of the transverse colon (Fig. 4b). The mesentery was divided to include the LCA aneurysms. Given her clinical condition, we elected to not perform a colorectal anastomosis, but instead performed a Hartmann’s procedure with an end transverse colon colostomy. The surgery took 145 min, and the estimated blood loss was 4.5 L. Packed red blood cell transfusion volume was approximately 1.96 L, and fresh frozen plasma volume was 1.44 L.

**Fig. 5:**
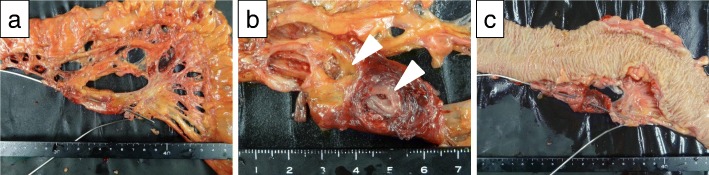
Macroscopic findings from surgery. **a** Gross specimen of the left colic artery (LCA) aneurysm. A wire was passed through the left colic artery. **b** Magnified view of the LCA aneurysms. A 2-cm cystic lesion and 1-cm cystic lesion were identified in the left colic artery (white arrowheads). No thrombus was identified. **c** Gross specimen of the left colon. No ischemic changes and no tumor were observed in the excised colon

**Fig. 6:**
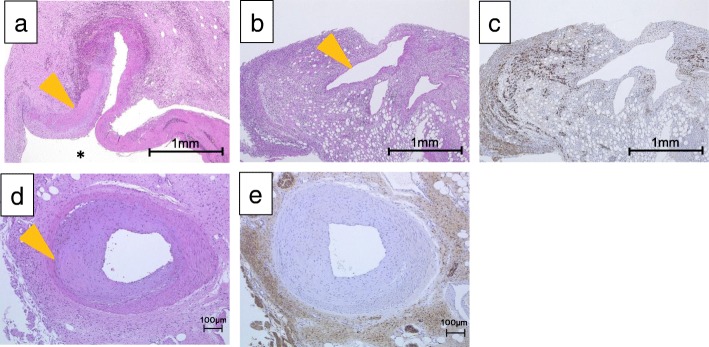
Histopathological findings. **a** The fibrous tissue of the left colic artery (LCA) aneurysm stained with hematoxylin and eosin stain (× 40). The orange arrowhead indicates the arterial wall. The asterisk indicates the lumen. **b**, **c** The fibrous tissue of the left colic artery (LCA) aneurysm as stained with hematoxylin and eosin (× 40). The orange arrowhead indicates the vein (**b**). The fibrous tissue of the LCA aneurysm showed high cytoplasmic expression of S-100 (× 40) (**c**). Neurofibroma from NF1 was seen around the vein and adipose tissue near the LCA. **d**, **e** The fibrous tissue around the vessels stained with hematoxylin and eosin (× 100). The orange arrowhead indicates vessels near the LCA (**d**). The fibrous tissue around the vessels showed high cytoplasmic expression of S-100 (× 100) (**e**)

In reviewing the specimens, a 2-cm aneurysmal sac and 1-cm aneurysmal sac were identified in the LCA. No thrombus was identified (Fig. 5a, b). No ischemic changes and no tumor were observed in the excised colon (Fig. 5c). Interestingly, fibrous tissue around the left colic artery stained positive for S-100 protein, suggesting that neurofibroma from NF1 might have been associated with the rupture of these aneurysms due to weakened integrity of the vascular walls (Fig. 6a–e). A follow-up abdominal CT showed no recurrence of the endoleak. The patient was discharged 37 days after the last operation without any other post-operative complications.

## Discussion

While vascular abnormalities associated with NF1 are rare, there are some reports of vascular complications related to NF1. In their series of 22 cases from 1978 to 2016, Uneda et al. characterized the presence of extracranial vertebral artery aneurysms associated with NF1 [20]. Conforti et al. detected intracranial aneurysms in two of 39 patients with NF1 [21]. De Santis et al. also described a radial artery aneurysm associated with NF1 [22]. These studies describe the diagnosis and treatment of a primary aneurysm in a variety of sites. In contrast, our case is atypical in that we describe rupture of secondarily formed LCA aneurysms.

In analyzing the mechanism of the LCA aneurysms, it is essential to consider the disease of NF1 itself. Arterial lesions associated with NF1 are classified into two groups based on the diameter of the vessels [23]. In the larger vessel groups, including the aorta and carotid arteries, the increase of neurofibromatous or ganglioneuromatous tissue, which surround the vessel, can cause the degeneration of the vascular intima and vascular media, leading to the arterial aneurysm. On the other hand, in the small vessel group, the degeneration of vascular smooth muscle and elastomer fibers and artery fibrosis directly contributed to artery dysplasia. Leier et al. also suggested that the increase of neurofibromatous or ganglioneuromatous tissue generates increased pressure in the peri-vascular vessels that supply large vessels such as the aorta, leading to ischemia of the large blood vessel walls resulting in aneurysm [24]. Similar to these cases, we confirmed an increase of neurofibromatous tissue around the left colic artery aneurysms. There was no observed atherosclerosis as a cause of the AAA.

**Fig. 7:**
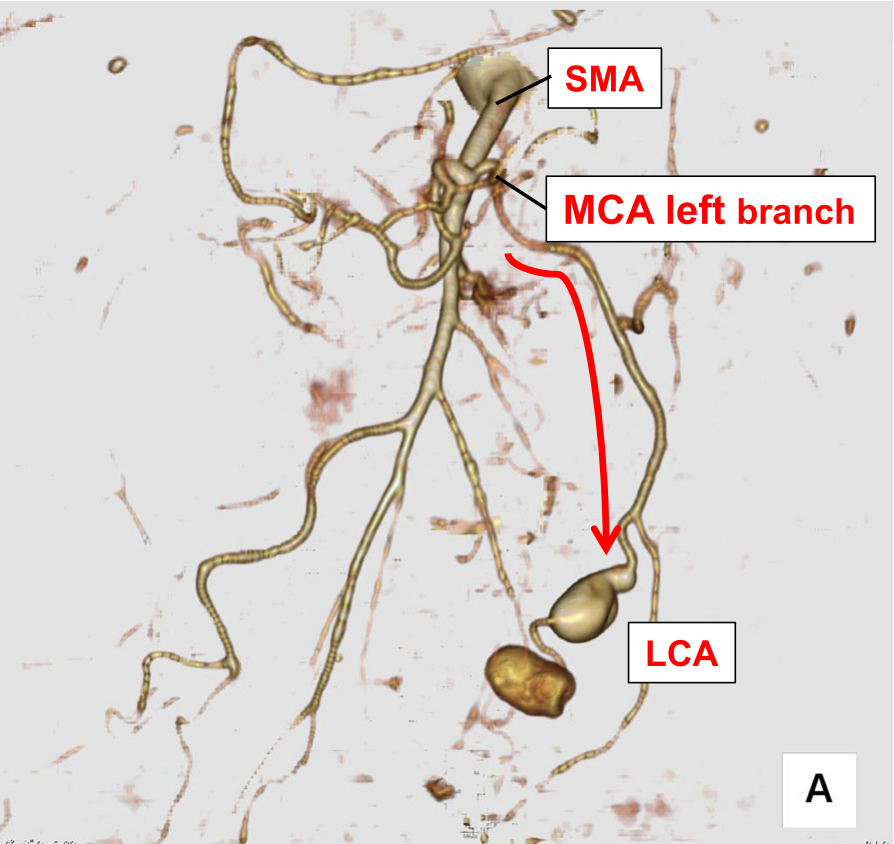
A 3D-CT angiography showing two sequential aneurysms in the left colic artery (LCA). The blood flow of the left colic artery was derived from the middle colic artery (MCA) left branch via the superior mesenteric artery (SMA) (red line)

To determine the cause of the LCA aneurysm rupture, which occurred shortly after the patient’s index operation, it is also important to consider the blood flow dynamic and how it changed following the patient’s treatment for her ruptured AAA. Before the LCA aneurysms developed, EVAR was performed twice for the AAA. While the risks of EVAR, including intestinal ischemia and necrosis, are lower than open surgery [25–27], the stent graft treatment for AAA by EVAR can decrease IMA flow and lead to intestinal necrosis [28]. In the present case, an abdominal three-dimensional-CT (3D-CT) angiography revealed a defect in the IMA caused by EVAR, and blood flow to the LCA was subsequently supplied by the middle colic artery (MCA) left branch via the superior mesenteric artery (SMA) (Fig. 7). Ohno et al. confirmed a defect of the IMA in 25 of 1000 cases by 3D-CT angiography, concluding that the blood flow of the MCA and the internal iliac artery (IIA) increased in order to compensate for the IMA deficit [29]. Thus, the IMA obstruction may have caused changes to the regional blood flow and increased related arterial pressures, resulting in the formation of new artery aneurysms in the LCA. Taken together, the weakness of the arterial vessel wall inherent to NF1, and the blood flow change caused by IMA deficit, may have resulted in these secondary LCA aneurysms. Given that the number and location of NF1-related aneurysms are different from non-NF1-related aneurysms [30] and that NF1 aneurysms can be caused without hypertension [31], the primary etiologies of the LCA aneurysms may be the weakness of vessel wall inherent to NF1.

With many potential treatments available for arterial aneurysms, the treatment should be tailored to the patient’s underlying conditions and diseases. Moreover, aneurysms related to NF1 should be treated immediately to prevent rupture. In the present case, there was little time to prepare for the primary operation, and as a result, we performed EVAR twice. In the third operation, while coil embolization is usually considered first, we selected surgery. Coil embolization posed a significant technical difficulty due to two artery aneurysms located in sequential continuity within the left colic artery. In addition, the patient presented in extremis and in need of immediate intervention. Lastly, given her history of NF1, the integrity of coil embolization would be suspect in the setting of her diseased vessels. A left hemicolectomy was indicated for the surgical intervention due to the hematoma caused by the past abdominal aortic rupture and perivascular inflammation. Leaving the left colon and removing only the left colic aneurysms were considered to be of prohibitive risk in this case, as the blood flow to the left colon would have been significantly compromised. Insufficient blood flow is known to increase the possibility of intestinal necrosis and anastomotic leakage [32]. Resection to the middle of the transverse colon was performed to minimize ischemia at Griffiths’ point, a known watershed area of vascular flow [33]. Due to her hemodynamic instability and resuscitation, we elected to perform an end colostomy as a colorectal anastomosis would have a high risk of leak. Although indocyanine green (ICG) fluorescence as a means to evaluate colon viability [34] was considered, it was not performed since we did not have access to ICG during the emergency surgery in our institution at the time.

## Conclusions

EVAR appears to be a safe approach for NF1 patients with AAA due to the extreme fragility of the vessel wall. However, endovascular management can be challenging at times, such as the case with our patient. Thus, the surgeon should be ready to consider open surgery in the settings of underlying connective tissue disease or emergency circumstances.

## Abbreviations

3D-CT: Three-dimensional-CT
AAA: Abdominal aortic aneurysm
CT: Computed tomography
EVAR: Endovascular aortic repair
ICG: Indocyanine green
IIA: Internal iliac artery
IMA: Inferior mesenteric artery
IVC: Inferior vena cava
LCA: Left colic artery
MCA: Middle colic artery
NF1: Neurofibromatosis type 1
SMA: Superior mesenteric artery
SRA: Superior rectal artery
